# Supplementary material for: Data-Driven and Machine Learning to Screen Metal–Organic Frameworks for the Efficient Separation of Methane
Source: Nanomaterials (Basel). 2024 Jun 24;14(13):1074. doi: 10.3390/nano14131074 (PMC11243175; doi:10.3390/nano14131074)
Supplement: Supplementary file 1 [file nanomaterials-14-01074-s001.zip › nanomaterials-3052630-supplementary.pdf]

Supporting Information

Data-Driven and Machine Learning to Screen Metal–Organic Frameworks for the Efficient Separation of Methane

Yafang Guan [1], Xiaoshan Huang [1], Fangyi Xu [1], Wenfei Wang [1], Huilin Li [1], Lingtao Gong [1], Yue Zhao [2], Shuya Guo [1,*], Hong Liang [1,*] and Zhiwei Qiao [1,*]

[1] Guangzhou Key Laboratory for New Energy and Green Catalysis, School of Chemistry and Chemical Engineering, Guangzhou University, Guangzhou 510006, China
[2] State Key Laboratory of NBC Protection for Civilian, Beijing 100191, China

Table of contents:

Section

S1 Adsorbate Force Field Parameters 2

S2 Characteristics of gas molecules 5

S3 Details of Model Training 6

S4 Analysis of each single variable... 16

S5 Evaluation of machine learning 19

S6 Analysis of the relative importance of features 23

S7 Various structural parts of MOF... 24

References 26

Section S1. Adsorbate Force Field Parameters

Table S1. Lennard–Jones parameters of MOF[47]

Atom	ε/k_B [K]	σ [Å]	Atom	ε/k_B [K]	σ [Å]	Atom	ε/k_B [K]	σ [Å]
Ac	16. 60	3. 10	Ge	190. 69	3. 81	Po	163. 52	4. 20
Ag	18. 11	2. 80	Gd	4. 53	3. 00	Pr	5. 03	3. 21
Al	254. 09	4. 01	H	22. 14	2. 57	Pt	40. 25	2. 45
Am	7. 04	3. 01	Hf	36. 23	2. 80	Pu	8. 05	3. 05
Ar	93. 08	3. 45	Hg	193. 71	2. 41	Ra	203. 27	3. 28
As	155. 47	3. 77	Ho	3. 52	3. 04	Rb	20. 13	3. 67
At	142. 89	4. 23	I	170. 57	4. 01	Re	33. 21	2. 63
Au	19. 62	2. 93	In	301. 39	3. 98	Rh	26. 67	2. 61
B	90. 57	3. 64	Ir	36. 73	2. 53	Rn	124. 78	4. 25
Ba	183. 15	3. 30	K	17. 61	3. 40	Ru	28. 18	2. 64
Be	42. 77	2. 45	Kr	110. 69	3. 69	S	137. 86	3. 59
Bi	260. 63	3. 89	La	8. 55	3. 14	Sb	225. 91	3. 94
Bk	6. 54	2. 97	Li	12. 58	2. 18	Sc	9. 56	2. 94
Br	126. 29	3. 73	Lu	20. 63	3. 24	Se	146. 42	3. 75
C	52. 83	3. 43	Lr	5. 53	2. 88	Si	202. 27	3. 83
Ca	119. 75	3. 03	Md	5. 53	2. 92	Sm	4. 03	3. 14
Cd	114. 72	2. 54	Mg	55. 85	2. 69	Sn	285. 28	3. 91
Ce	6. 54	3. 17	Mn	6. 54	2. 64	Sr	118. 24	3. 24
Cf	6. 54	2. 95	Mo	28. 18	2. 72	Ta	40. 75	2. 82
Cl	114. 21	3. 52	N	34. 72	3. 26	Tb	3. 52	3. 07
Cm	6. 54	2. 96	Na	15. 09	2. 66	Tc	24. 15	2. 67
Co	7. 04	2. 56	Ne	21. 13	2. 66	Te	200. 25	3. 98

Cr	7. 55	2. 69	Nb	29. 69	2. 82	Th	13. 08	3. 03
Cu	2. 52	3. 11	Nd	5. 03	3. 18	Ti	8. 55	2. 83
Cs	22. 64	4. 02	No	5. 53	2. 89	TI	342. 14	3. 87
Dy	3. 52	3. 05	Ni	7. 55	2. 52	Tm	3. 02	3. 01
Eu	4. 03	3. 11	Np	9. 56	3. 05	U	11. 07	3. 02
Er	3. 52	3. 02	O	30. 19	3. 12	V	8. 05	2. 80
Es	6. 04	2. 94	Os	18. 62	2. 78	W	33. 71	2. 73
F	25. 16	3. 00	P	153. 46	3. 69	Xe	167. 04	3. 92
Fe	6. 54	2. 59	Pa	11. 07	3. 05	Y	36. 23	2. 98
Fm	6. 04	2. 93	Pb	333. 59	3. 83	Yb	114. 72	2. 99
Fr	25. 16	4. 37	Pd	24. 15	2. 58	Zn	62. 39	2. 46
Ga	208. 81	3. 90	Pm	4. 53	3. 16	Zr	34. 72	2. 78

Table S2. Lennard–Jones parameters and charges of adsorbates[48, 49]

Atom	ε/k_B [K]	σ [Å]	Charge (e)	Atom	ε/k_B [K]	σ [Å]	Charge (e)
C_CO_2	27.0	2.80	+0.700	S_H_2S	122.0	3.60	0
O_CO_2	79.0	3.05	–0.350	M_H_2S	0	0	–0.420
CH_4	148.0	3.73	0	H_H_2	0	0	+0.468
N_N_2	36.0	3.31	–0.482	com_H_2	36.7	2.96	–0.936
com_N_2	0	0	+0.964	He_He	10.9	2.64	0
O_O_2	49.0	3.02	–0.113	H_H_2S	50.0	2.50	+0.210
com _O_2	0	0	+0.226	CH_4	98.0	3.750	0

The molecular diffusion coefficients obtained through Molecular Dynamics (MD) simulations have been consistently validated against experimental data across numerous studies, demonstrating their precision.[50] As shown in Figure S1, the simulated values align closely with experimental findings, which substantiates the trustworthiness and effectiveness of the MD simulation technique for such applications.

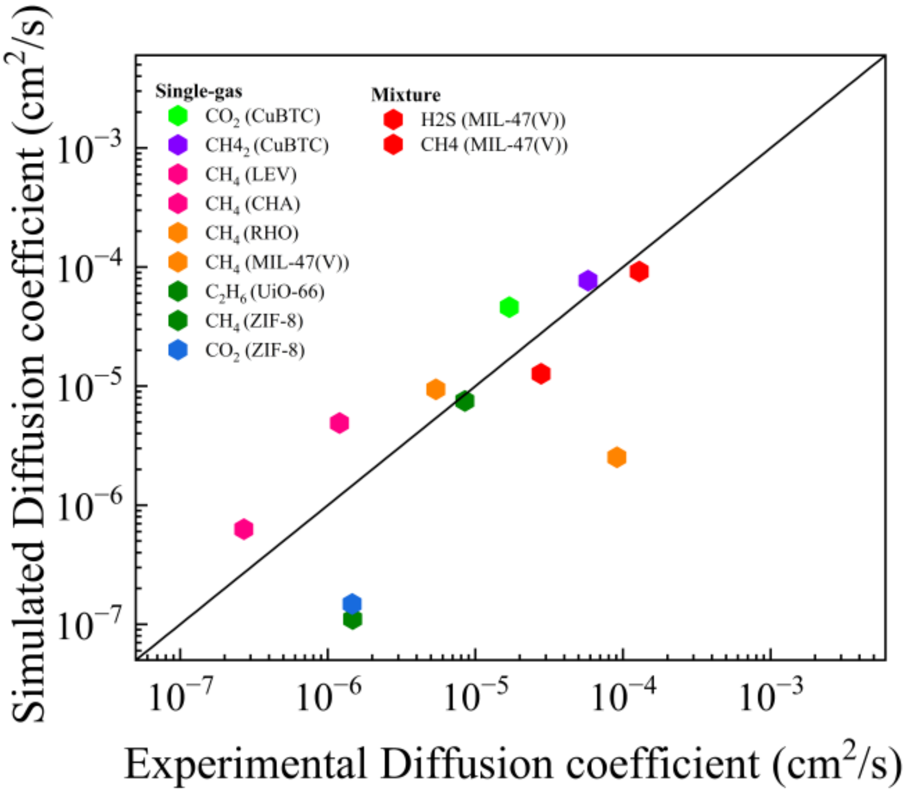

Figure S1. Comparison of simulated gas diffusivities and the experimental data for various MOF.[51]

Section S2. Characteristics of gas molecules

Table S3. Differences of kinetic diameter, polarizability, dipole moment, and quadruple moment between binary gas mixtures.

	Gas mixture *i/j*	*ΔDia* [Å]	*ΔPol* [$\times10^{25}$/cm^3]	*ΔDip* [$\times10^{18}$/esu cm]	*ΔQua* [$\times10^{26}$/esucm^2]
In Molecular Simulation	He/CH_4	1.2	23.88044	0	0
	H_2/CH_4	0.91	17.888	0	-0.662
	CO_2/CH_4	0.5	-3.18	0	-4.3
	O_2/CH_4	0.34	10.118	0	-0.39
	H_2S/CH_4	0.18	-12.73	-0.97833	0
	N_2/CH_4	0.16	8.527	0	-1.52

Table S4. Physical properties of gas molecules.[52]

Gas	Kinetic diameter [Å]	Polarizability [$\times10^{25}$ /cm^3]	Dipole moment [$\times10^{18}$ /esu cm]	Quadruple moment [$\times10^{26}$ /esu cm^2]
He	2.6	2.04956	0	0
H_2	2.89	8.042	0	0.662
CO_2	3.3	29.11	0	4.30
O_2	3.46	15.812	0	0.39
H_2S	3.62	37.82	0.97833	—
N_2	3.64	17.403	0	1.52
CH_4	3.758	25.93	0	0

Section S3. Details of Model Training

Python 3.9.12 was used for all training tasks.

Table S5. The version information of tool packages used for building ML model.

Package	Version
scikit-learn	1.0.2
numpy	1.21.5
random	1.2.2
pandas	1.3.5
shap	0.40.0
lightgbm	3.3.2
xgboost	1.1.2
joblib	1.1.0

Overview of four machine learning algorithms

In this study, we employ four distinct machine learning (ML) methods: Random Forest (RF), Gradient Boosting Regression Trees (GBRT), eXtreme Gradient Boosting (XGBoost), and Light Gradient Boosting Machine (LightGBM). Each of these algorithms is an application of the ensemble learning paradigm, which combines multiple models to improve predictive performance. Below, we outline the principal distinctions, along with the strengths and limitations of the four algorithms. (A summary of the following algorithms, excluding classification algorithms, is based on the introduction of the regression models used in this work.)

Random Forest (RF) Algorithm

Building upon the Bagging methodology and utilizing decision trees as its predictive engines, the Random Forest (RF) algorithm enhances the model by incorporating an element of randomness in the feature selection phase of each tree's training. The process can be succinctly divided into four key components, as depicted in Figure S2: The construction of a novel training sample set is achieved through the methodical extraction of repeated random samples of size k from the original training set, which comprises N samples. In the context of this study, features are selected in an arbitrary manner, and the model is trained on the entire spectrum of these features. Predictions from an individual decision tree are derived from the extracted samples, and these individual forecasts are subsequently amalgamated by averaging to yield the conclusive predictions. The random forest algorithm boasts several advantages, including its robust generalizability, its adeptness at managing datasets with missing values, and its utility without the need for data normalization. Nonetheless, it has a limitation: its predictive capacity is confined to the range encapsulated by the training set. This can lead to overfitting, especially when the algorithm is applied to noisy datasets that require more nuanced modeling.

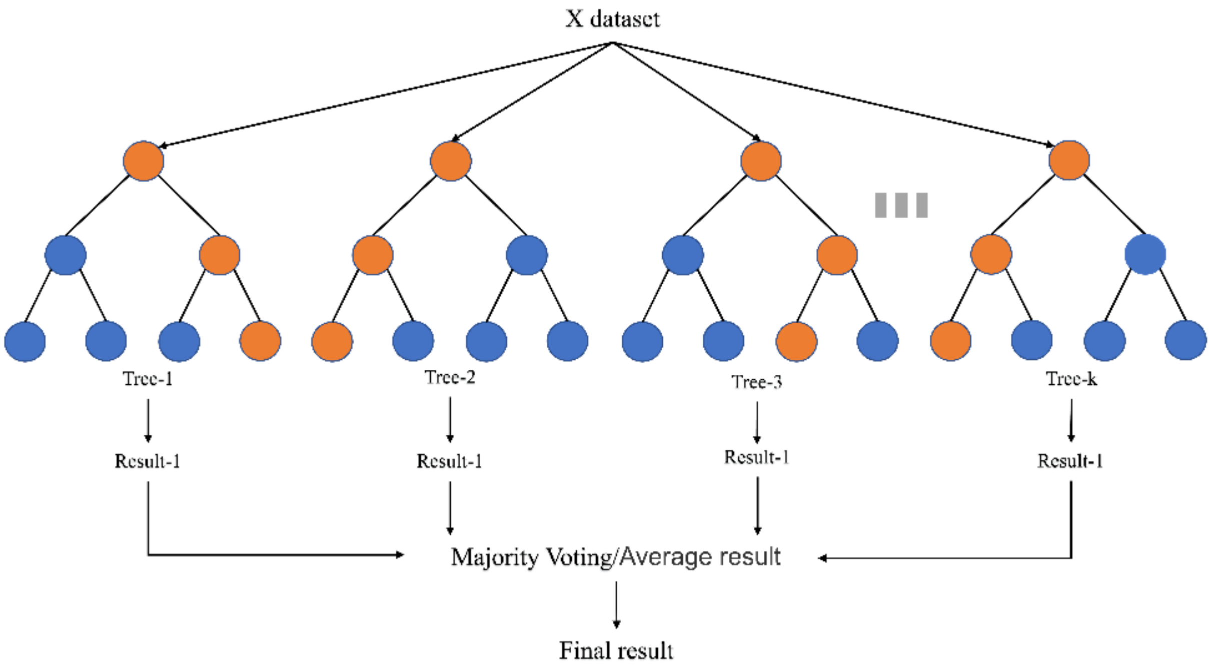

Figure S2. Random Forest model.

Gradient boosting regression tree Algorithm

Gradient boosting serves as the core principle of the Gradient Boosting Regression Tree (GBRT) approach. This method distinguishes itself from traditional boosting techniques by iteratively addressing the residuals of the previous model. Specifically, each new model is crafted to align with the gradient that minimizes these residuals, thereby refining the overall prediction with each iteration. The final iteration capitalizes on the gradient learning loss function to support the derivation of forecasts. As depicted in Figure S3, the "squared error" loss function utilized in this study ensures a smooth fitting of errors throughout the learning process. Successive regression trees are employed to refine the GBRT by assimilating the outcomes and residuals from preceding trees. GBRT's strengths are manifold. It exhibits flexibility in handling various data types and enhances predictive accuracy within a relatively short timeframe of parameter tuning. The performance of GBRT is further augmented when paired with RF, although this combination presents its own set of challenges. Training data in parallel becomes complex due to the inherent serial dependencies in the learner models, a consequence of the Boosting framework's architecture.

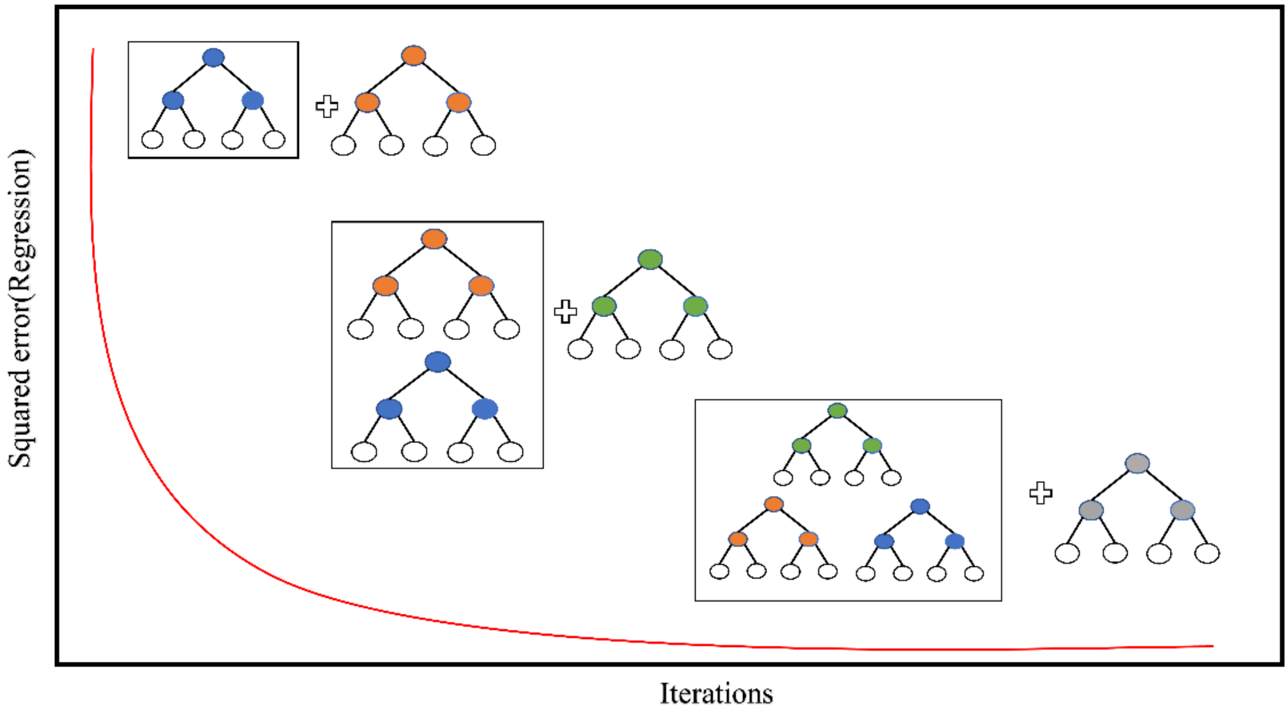

Figure S3. GBRT model.

Extreme Gradient Boosting Algorithm

Extreme Gradient Boosting, known as XGBoost, is an enhancement to the traditional Gradient Boosting Decision Tree (GBDT) algorithm, developed by Tianqi Chen from the University of Washington. XGBoost refines the approximation of residuals by employing the negative gradient of the model on the data, coupled with a Taylor series expansion of the loss function's residuals. Additionally, it incorporates a regularization term that accounts for model complexity. XGBoost outperforms GBDT by leveraging parallel processing capabilities of CPUs, which significantly speeds up the computation. The algorithm is designed to be scalable, with each iteration building upon the previous one efficiently (the cost of the t-th iteration function is priced with a factor of t-1 times the gradient of the predicted values).

XGBoost's next-generation capabilities also include data preprocessing through sorting and storing data in a block structure, which facilitates parallelism during training. Before splitting nodes, the algorithm systematically evaluates the gain for each feature and selects the one that yields the highest gain. This process can be conducted in parallel, with multiple threads determining the gain of different features.However, as illustrated in Figure S4, XGBoost employs a level-wise tree growth method, which processes all leaf nodes in the current layer uniformly. This approach can lead to the splitting of leaf nodes that may not yield significant profit, thus incurring unnecessary computational costs.

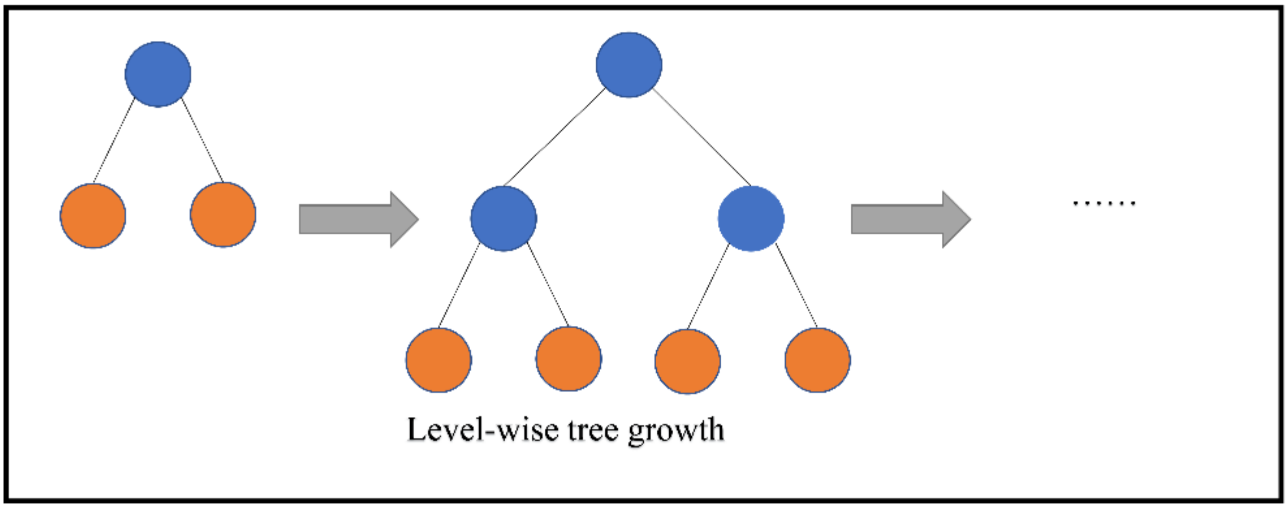

Figure S4. XGBoost model.

LightGBM Algorithm

Developed by Microsoft Research Asia, LightGBM is an algorithm that leverages the Gradient Boosting Decision Tree (GBDT) framework to enhance computational efficiency, particularly for tackling the challenges of big data predictions. LightGBM incorporates two innovative methods: Gradient-based One-Side Sampling (GOSS) and Exclusive Feature Bundling (EFB), which are employed for efficient random sampling and feature extraction, respectively. EFB streamlines the feature selection process by not scanning all features to identify the optimal split point. Instead, it reduces dimensionality by intelligently grouping features, thereby minimizing the computational cost associated with finding the best split. Meanwhile, GOSS refines the gradient calculation by selectively sampling data points, which not only maintains accuracy but can also enhance it in certain scenarios while significantly reducing the time required for processing.

LightGBM also employs a Histogram-based algorithm to effectively integrate exclusive features. The essence of this technique involves discretizing continuous feature values into k distinct intervals and constructing a histogram with a width of k, as illustrated in Figure S5(b). The histogram accumulates data statistics using these discretized values as indices, which are then used to identify the optimal split points. This approach substantially reduces both computational cost and memory usage. While XGBoost's default pre-sorted algorithm demands O(#data) computations, LightGBM's Histogram algorithm operates with O(#bins) computations, where #bins is typically much smaller than #data.Figure S5(a) illustrates that LightGBM adopts a

leaf-wise growth strategy, in contrast to XGBoost's level-wise strategy. By identifying and splitting the leaf with the highest potential gain, LightGBM can achieve greater accuracy with fewer mistakes, given the same number of splits. However, the leaf-wise approach may lead to overfitting with small sample sizes. To counteract this, LightGBM offers the option to set a maximum depth (Max depth) for the trees, which helps to prevent overfitting.

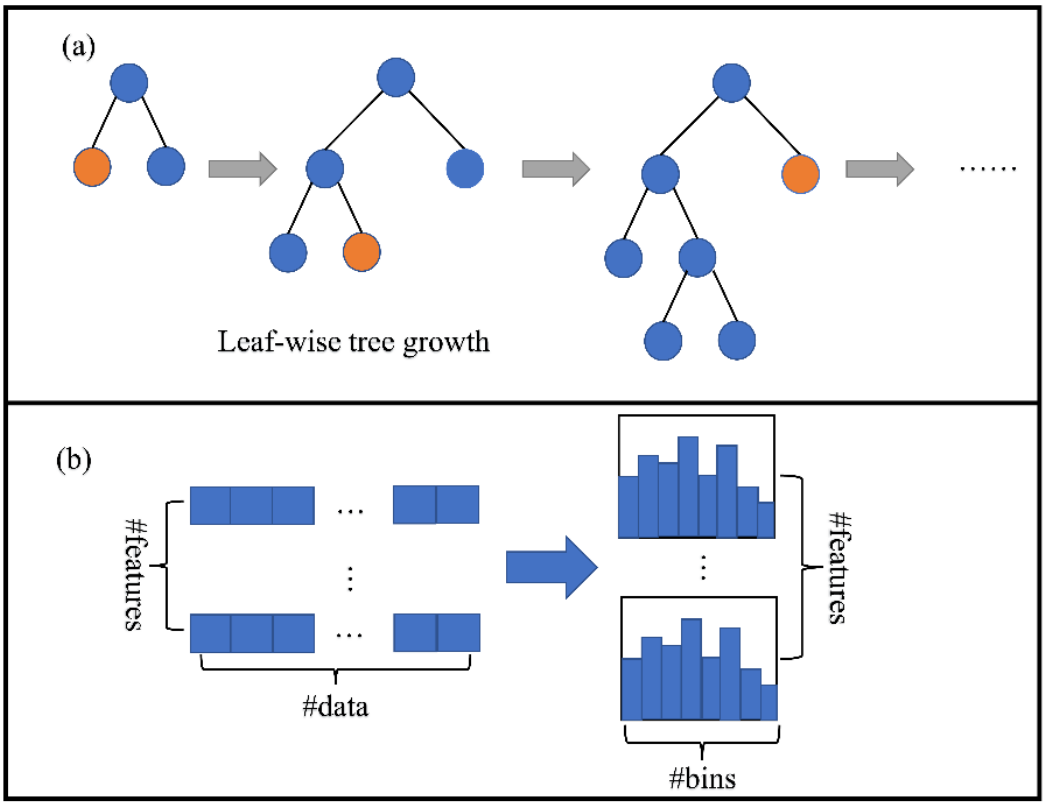

Figure S5. LightGBM model.

Table S6. Hyperparameters set in machine learning methods.

Model	Hyperparameter	Value (On *D*)	Value (On S_{diff})
RF	n_estimators	800	800
	max_depth	14	14
	random_state	42	42
	criterion	'squared_error'	'squared_error'
GBRT	learning_rate	0.1	0.1
	loss	'squared_error'	'squared_error'
	n_estimators	600	800
	subsample	1	1
	criterion	'friedman_mse'	'friedman_mse'
	max_depth	10	12
	alpha	0.8	0.5
	verbose	0	0
	max_leaf_nodes	None	None
	warm_start	False	False
XGBoost	n_estimators	600	650
	max_depth	12	14
	min_child_weight	1	1
	subsample=0.8	0.8	0.8
	gamma=0.0	0	0
	colsample_bytree	0.8	0.8
	nthread	None	None
	reg_alpha	0.8	0.8
	reg_lambda	1	1
	seed	1314	1314

	n_jobs	-1	-1
LightGBM	objective	'regression'	'regression'
	n_estimators	620	500
	learning_rate	0.1	0.05
	num_leaves	420	500
	force_col_wise	True	True
	colsample_bytree	0.8	0.8
	subsample_for_bin	50000	220000
	random_state	1314	100
	n_jobs	-1	-1
	min_child_samples	5	5
	reg_alpha	0.6	0
	reg_lambda	0.7	0

Algorithm Evaluation Index

In this work, the performance of each ML algorithm was evaluated by calculating the R^2 values and the root-mean-square error (RMSE). The R^2 value was calculated using eq. S1, where n, y_i, u_i, and $\bar{u}$ are the number of MOFs, the simulated diffusion coefficient of gas molecule (diffusion selectivity of ideal binary gas), the predicted diffusion coefficient of gas molecule (diffusion selectivity of ideal binary gas) and average diffusion coefficient of gas molecule (diffusion selectivity of ideal binary gas), respectively. The various error values for each algorithm were calculated using eq. S2.

In this study, we assessed the performance of each machine learning (ML) algorithm by determining their R^2 values and root-mean-square errors (RMSE). The R^2 value, a measure of how well the model fits the data, was computed using equation S1. This equation takes into account the number of metal-organic frameworks (MOFs) (n), the simulated diffusion coefficient of the gas molecule (representing the diffusion selectivity of an ideal binary gas) (y_i), the predicted diffusion coefficient of the gas molecule (u_i), and the average diffusion coefficient of the gas molecule ($\bar{u}$). The RMSE, which provides a measure of the average error between the predicted and actual values, was calculated for each algorithm using equation S2. This metric offers insight into the typical magnitude of the errors made by the model during predictions. By employing these quantitative metrics, we were able to rigorously evaluate and compare the predictive accuracy and reliability of the different ML algorithms utilized in this research.

$$R^2=1-\frac{\sum_{i=1}^{n}(y_i-u_i)^2}{\sum_{i=1}^{n}(y_i-\bar{u}_i)^2} \quad \text{(S1)}$$

$$\text{RMSE}=\sqrt{\frac{\sum_{i=1}^{n}(y_i-u_i)^2}{n}} \quad \text{(S2)}$$

k-fold cross-validation

The need for a separate validation set becomes redundant when employing cross-validation, with the test set reserved for the model's ultimate evaluation. This study employs a method known as k-fold cross-validation (k-fold CV), which segments the training set into k smaller subsets. As depicted in Figure S6, the process involves designating one of the k "folds" for validation, utilizing a subset of k-1 for training the model, and then applying the remaining data to verify the model's performance from the prior phase—akin to employing a test set to gauge the model's accuracy. The aggregated results from the k-fold cross-validation, calculated as the mean of the outcomes across all stages, provide an estimate of the model's overall performance.[0] For the purposes of this research, a 10-fold cross-validation approach has been implemented.

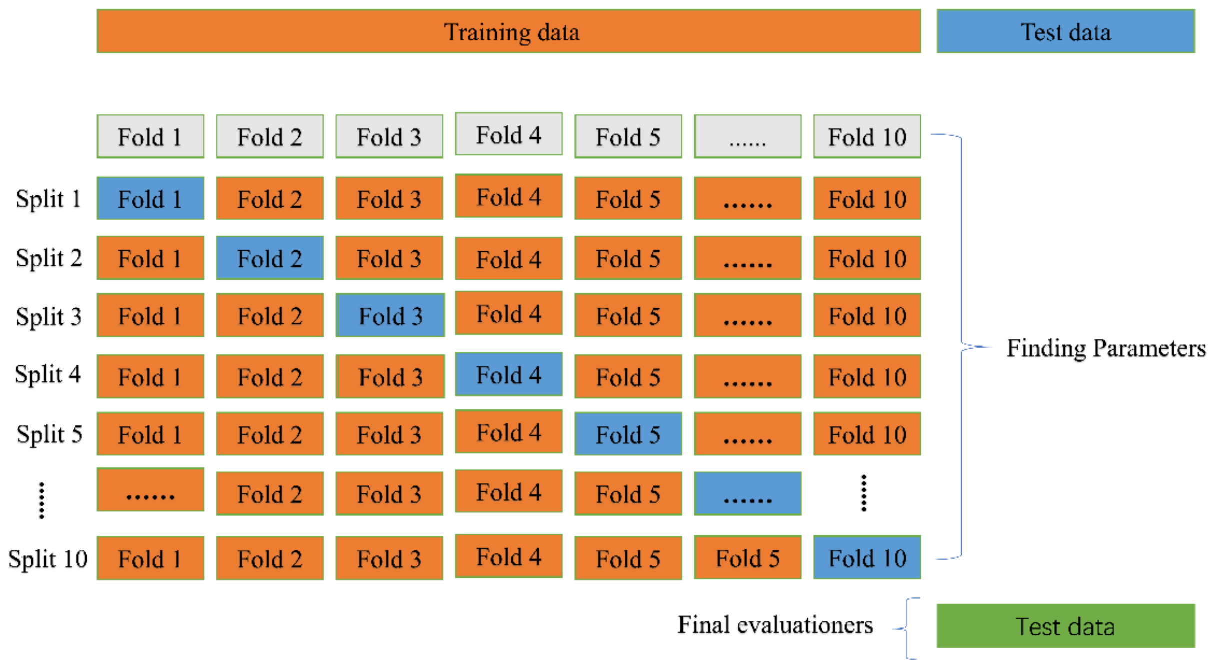

Figure S6. 10-fold cross-validation.

Section S4. Univariate analysis

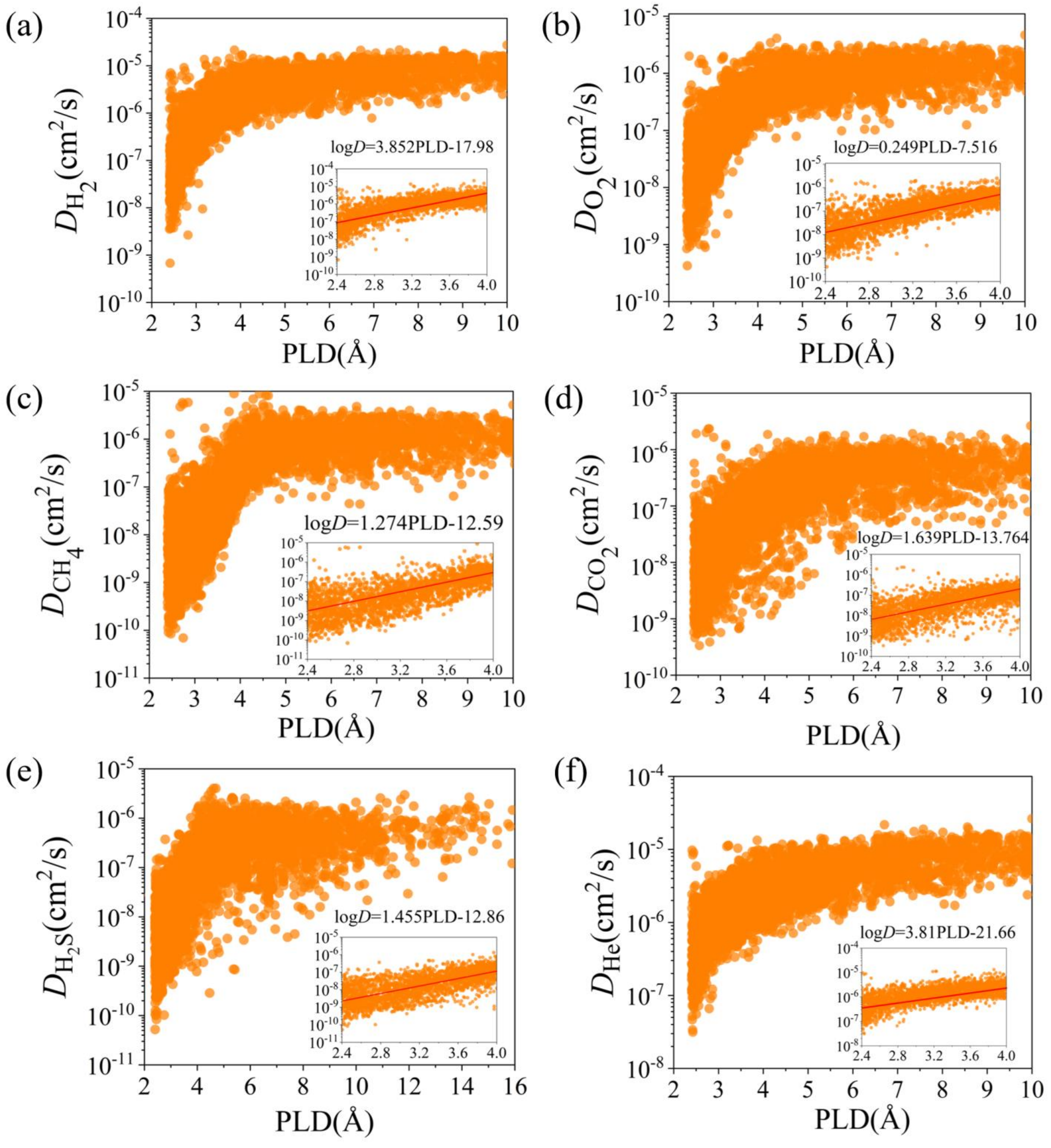

Figure S7. Gas diffusivity changes with PLD.

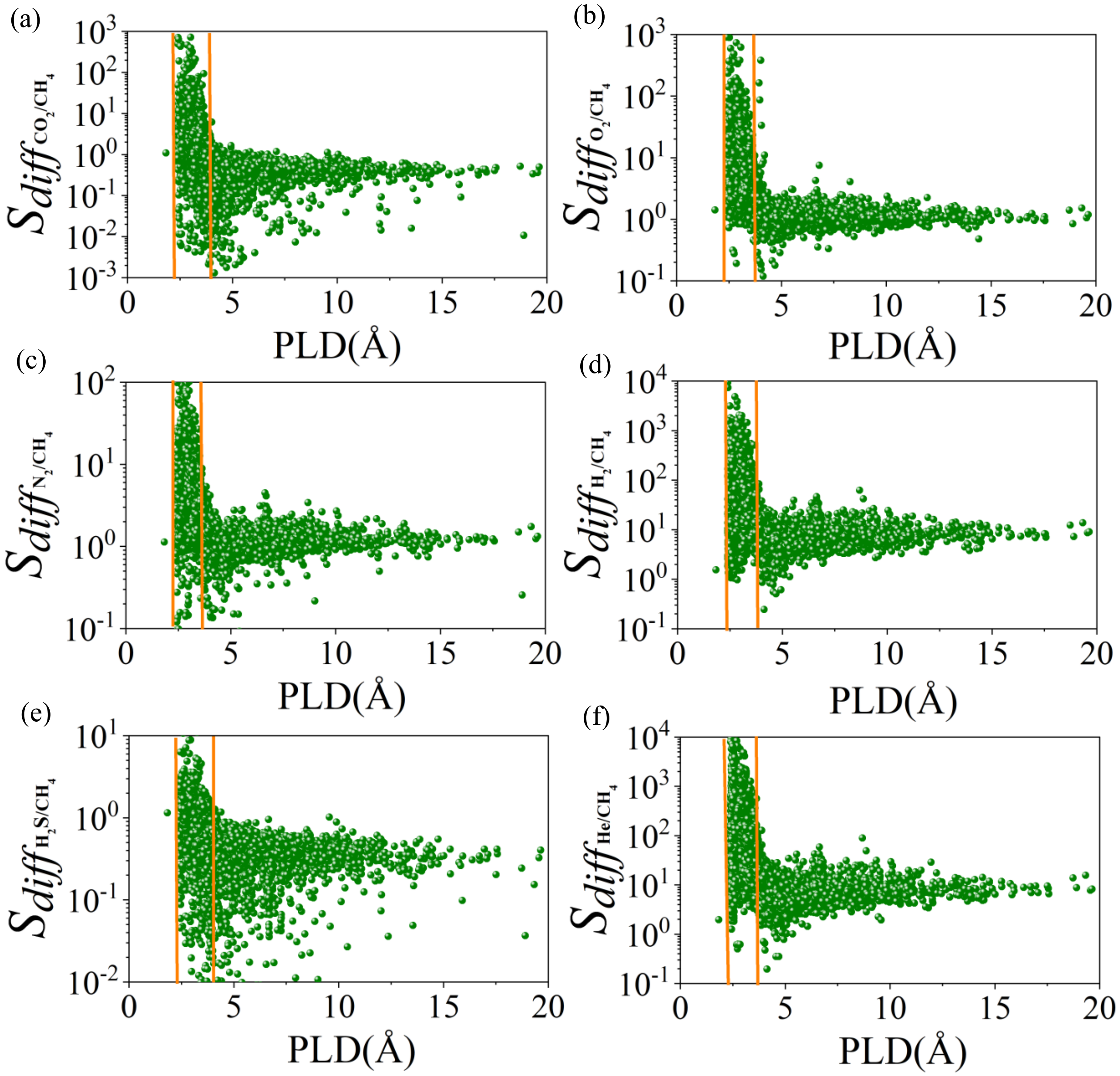

Figure S8. Gas diffusion selectivity varies with PLD.

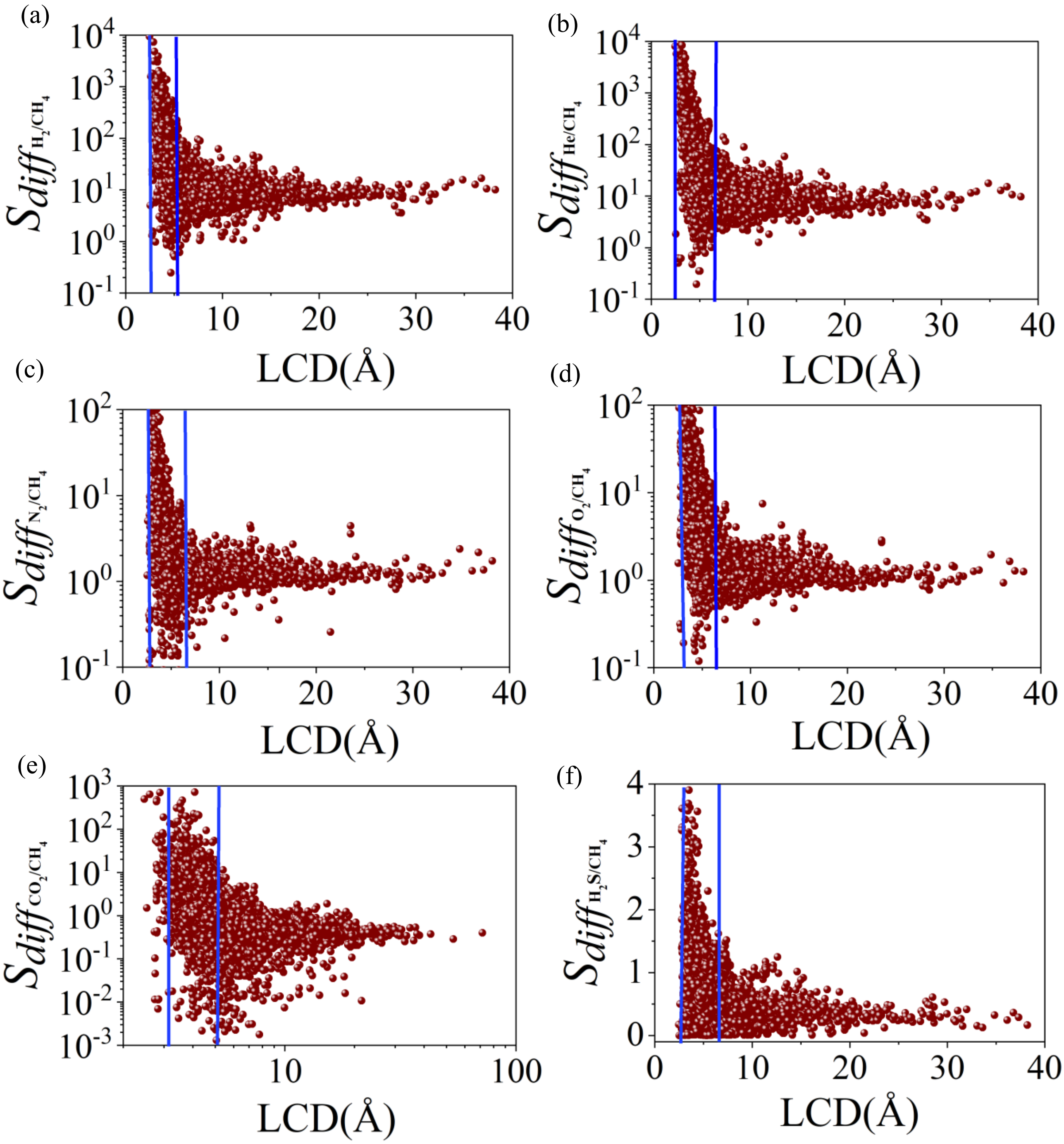

Figure S9. Gas diffusion selectivity varies with LCD.

Section S5. Evaluation of machine learning

Table S7. Evaluation of four algorithms for *D* and S_{diff}.

Algorithm	Performance	Indicators	Training set			Test set		
			R^2	MAE	RMSE	R^2	MAE	RMSE
RF	*D*	D_{CH_4}	0.987	0.078	0.115	0.918	0.200	0.289
		D_{O_2}	0.983	0.062	0.090	0.875	0.167	0.244
		D_{N_2}	0.984	0.071	0.105	0.890	0.186	0.276
		D_{H_2}	0.983	0.055	0.086	0.872	0.145	0.230
		D_{H_2S}	0.977	0.092	0.132	0.832	0.248	0.357
		D_{He}	0.981	0.043	0.063	0.871	0.113	0.161
		D_{CO_2}	0.963	0.097	0.141	0.748	0.255	0.369
		All	0.991	0.055	0.091	0.934	0.151	0.247
	S_{diff}	S_{O_2/CH_4}	0.969	0.051	0.082	0.767	0.136	0.217
		S_{N_2/CH_4}	0.942	0.048	0.084	0.561	0.125	0.208
		S_{H_2/CH_4}	0.968	0.071	0.107	0.781	0.180	0.271
		S_{H_2S/CH_4}	0.919	0.064	0.109	0.438	0.168	0.274
		S_{He/CH_4}	0.976	0.073	0.109	0.849	0.182	0.271
		S_{CO_2/CH_4}	0.940	0.098	0.153	0.586	0.247	0.384
		All	0.985	0.057	0.097	0.887	0.155	0.264
LGBM	*D*	D_{CH_4}	0.994	0.054	0.079	0.910	0.209	0.303
		D_{O_2}	0.988	0.055	0.076	0.865	0.173	0.254
		D_{N_2}	0.991	0.056	0.079	0.877	0.192	0.292
		D_{H_2}	0.988	0.049	0.071	0.864	0.151	0.238
		D_{H_2S}	0.990	0.064	0.087	0.815	0.259	0.374
		D_{He}	0.978	0.050	0.069	0.859	0.118	0.169

		D_{CO_2}	0.986	0.062	0.087	0.717	0.270	0.390
		All	0.993	0.052	0.077	0.954	0.127	0.207
	S_{diff}	S_{O_2/CH_4}	0.979	0.048	0.067	0.817	0.131	0.194
		S_{N_2/CH_4}	0.962	0.045	0.067	0.644	0.123	0.200
		S_{H_2/CH_4}	0.985	0.054	0.074	0.792	0.183	0.266
		S_{H_2S/CH_4}	0.957	0.054	0.080	0.405	0.1778	0.282
		S_{He/CH_4}	0.988	0.056	0.076	0.852	0.187	0.266
		S_{CO_2/CH_4}	0.982	0.059	0.083	0.592	0.254	0.386
		All	0.991	0.054	0.077	0.931	0.127	0.207
XGBoost	D	D_{CH_4}	0.999	0.026	0.038	0.912	0.207	0.299
		D_{O_2}	0.997	0.026	0.036	0.872	0.170	0.248
		D_{N_2}	0.987	0.027	0.039	0.890	0.186	0.267
		D_{H_2}	0.997	0.025	0.036	0.874	0.149	0.229
		D_{H_2S}	0.998	0.027	0.038	0.817	0.256	0.372
		D_{He}	0.994	0.025	0.035	0.865	0.115	0.164
		D_{CO_2}	0.997	0.027	0.039	0.738	0.263	0.383
		All	0.998	0.029	0.041	0.957	0.119	0.199
	S_{diff}	S_{O_2/CH_4}	0.996	0.021	0.029	0.838	0.120	0.182
		S_{N_2/CH_4}	0.993	0.020	0.030	0.668	0.114	0.181
		S_{H_2/CH_4}	0.997	0.022	0.030	0.809	0.172	0.250
		S_{H_2S/CH_4}	0.996	0.021	0.030	0.838	0.120	0.182
		S_{He/CH_4}	0.998	0.022	0.030	0.870	0.172	0.244
		S_{CO_2/CH_4}	0.997	0.021	0.031	0.561	0.246	0.376
		All	0.998	0.024	0.033	0.928	0.123	0.210
GBRT	D	Average	0.999	0.004	0.006	0.947	0.129	0.222
	S_{diff}	Average	0.999	0.005	0.007	0.907	0.135	0.240

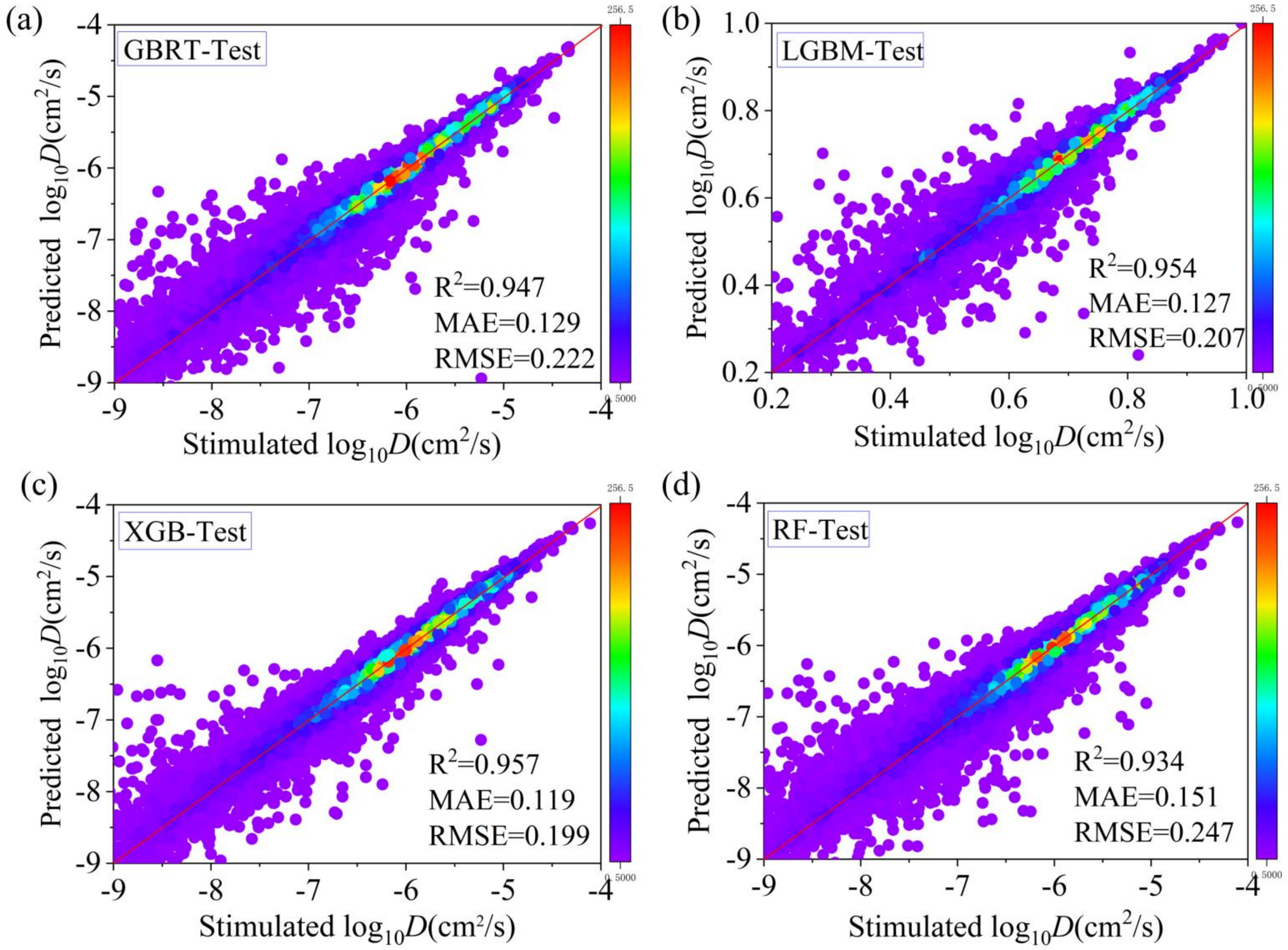

Figure S10. Predicted results of *D* by RF, XGB and GBRT ML algorithm models *versus* simulated results of CoRE-MOFs on the testing set.

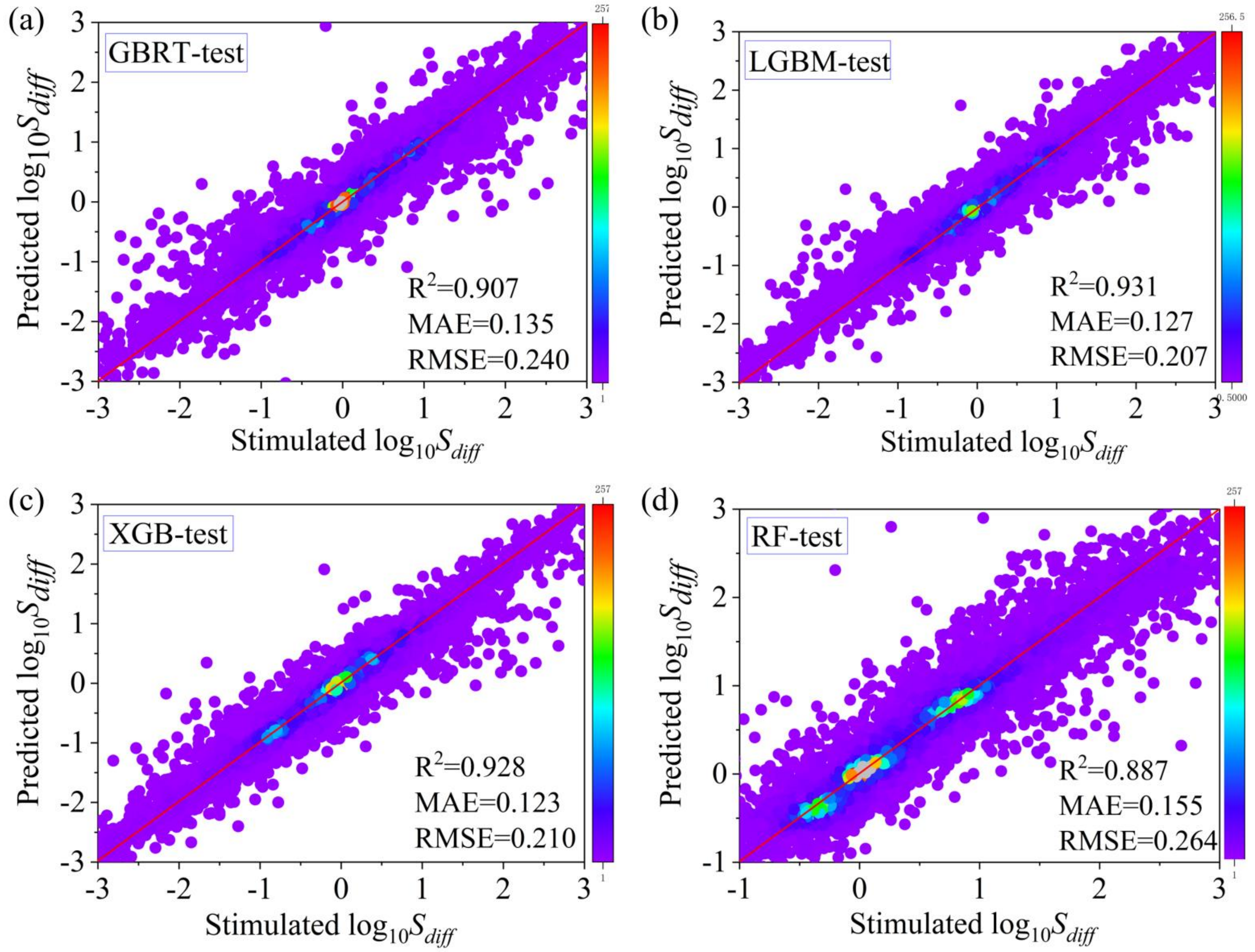

Figure S11. Predicted results of S_{diff} by RF, XGB and GBRT ML algorithm models *versus* simulated results of CoRE-MOFs on the testing set.

Section S6. Analysis of the relative importance of features

In this study, we utilized TreeExplainer in conjunction with Shapley Additive exPlanations (SHAP) to interpret the predictions made by a machine learning (ML) model. By integrating the LGBM model with SHAP values, we assessed the relative importance of each feature—in other words, the extent to which each feature impacts the model's output. The significance of each feature's influence is reflected by the average absolute SHAP value across the entire dataset. The outcomes of this analysis are presented in Tables S8 and S9.

Table S8. Importance ranking of features (Based on *D*).

No.	1	2	3	4	7	8	9
Feature	PLD	Dia_i	Pol_i	VSA	Qua_i	ρ	Dip_i
Importance (%)	36.13	-30.55	-30.82	21.67	0.50	-0.90	0.13

Table S9. Importance ranking of features (Based on S_{diff}).

No.	1	2	3	4	5	6	7	8	9	10	11	12	13
Feature	ΔPol	PLD	Dia_i	VSA	ΔDia	Pol_i	LCD	ϕ	ρ	ΔQua	ΔDip	Qua_i	Dip_i
Importance (%)	0.68	-23.61	-35.79	-14.84	0.36	-29.78	6.15	4.23	-9.47	2.13	-0.08	0	0

Shapley additive explanation

In this research, we employ SHAP to elucidate the significance and function of various predictors within our analysis. SHAP, grounded in game theory, treats the model's predicted values as an aggregation of contributions from each input feature. When approximating the original model f for a particular input x, the explanation's attribution values ϕ_i for each feature *i* should sum up to the output *f(x)*, represented by equation S3:

$$f(x)=\phi_0(f)+\sum_{i=1}^{M}\phi_i(f,x) \quad \text{(S3)}.$$

Where the sum of the feature attributes$\varphi_i(f,x)$ matches the output *f(x)* of the original model, M is the total number of input features, ϕ_0 represents the expected value when all inputs are missing, and ϕ_i is a measure of

the contribution of a given feature i to the prediction. According to game theory, the Shapley value is the only criterion that satisfies local accuracy, missing, and consistency. They are also very intuitive because they use the same units as the model output (D or S_{diff} in this work).

SHAP value is the Shapley value of a conditional expectation function $f(x)$, which can be derived from equation S4:

$$\phi_i = \sum_{R \in \mathcal{R}} \frac{1}{M!} [f_x(P_i^R \cup i) - f_x(P_i^R)] \quad \text{(S4)},$$

where $\mathcal{R}$ is the set of all feature orderings, P_i^R is the set of all features that come before feature i in ordering $\mathcal{R}$, and M is the number of input features for the model. For tree-based models, our study utilizes the TreeExplainer algorithm developed by Lundberg et al.[21], which adeptly calculates the SHAP values. The TreeExplainer assigns SHAP values to each individual sample within the dataset, providing a measure of the impact of each feature on the model's output. Subsequently, these individual predictions are aggregated and visualized to offer a comprehensive, global interpretation of the model's behavior.

Section S7. Various structural parts of MOFs

Table S10. Benchmark of D_i and $S_{diff\,(i/j)}$ for six gas mixtures

Gas mixture *i/j*	D_i	$S_{diff\,(i/j)}$	Gas mixture *i/j*	D_i	$S_{diff\,(i/j)}$
He/CH_4	9.50E-07	1000	O_2/CH_4	1.00E-07	26
H_2/CH_4	1.00E-06	300	N_2/CH_4	1.00E-07	5.5
CO_2/CH_4	9.00E-08	10	H_2S/CH_4	1.00E-09	1.9

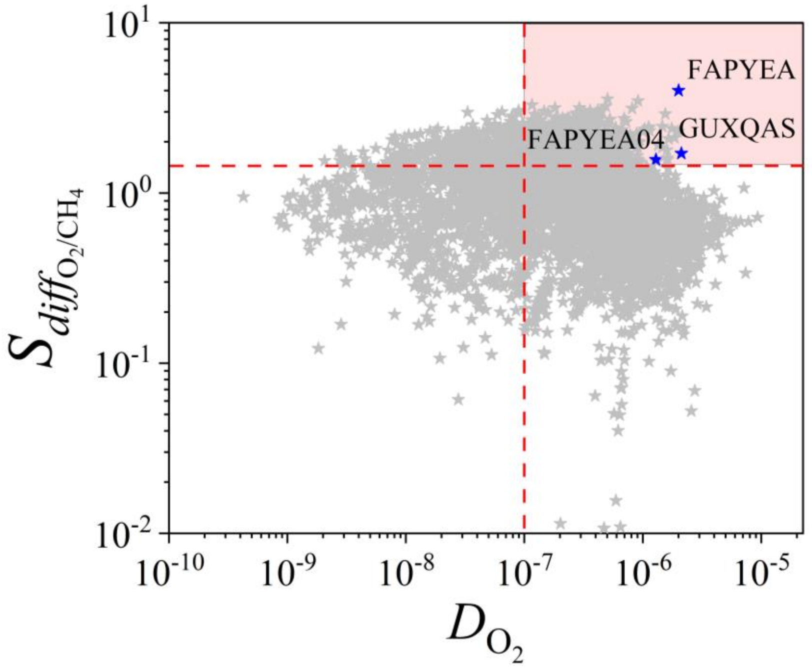

Figure S12. Example of Optimal Material Screening Strategy

References

[47] Rappé, A. K.; Casewit, C. J.; Colwell, K. S.; Goddard. III, W. A.; Skid, W. M., UFF, a Full Periodic Table Force Field for Molecular Mechanics and Molecular Dynamics Simulations. J. Am. Chem. Soc. 1992, 114 (25), 10024-10039.

[48] Martin, M. G.; Siepmann, J. I., Transferable Potentials for Phase Equilibria. 1. United-Atom Description of n-Alkanes. J. Phys. Chem. B 1998, 102 (14), 2569-2577.

[49] Shah, M. S.; Tsapatsis, M.; Siepmann, J. I., Development of the Transferable Potentials for Phase Equilibria Model for Hydrogen Sulfide. J. Phys. Chem. B 2015, 119 (23), 7041−7052.

[50] Pillai, R. S.; Jobic, H.; Koza, M. M.; Nouar, F.; Serre, C.; Maurin, G.; Ramsahye, N. A., Diffusion of Carbon Dioxide and Nitrogen in the Small-Pore Titanium Bis(phosphonate) Metal-Organic Framework MIL-91 (Ti): A Combination of Quasielastic Neutron Scattering Measurements and Molecular Dynamics Simulations. ChemPhysChem 2017, 18 (19), 2739-2746.

[51] Polat, H. M.; Zeeshan, M.; Uzun, A.; Keskin, S., Unlocking CO_2 Separation Performance of Ionic Liquid/CuBTC Composites: Combining Experiments with Molecular Simulations. Chem. Eng. J. 2019, 373, 1179-1189.

[52] Li, J. R.; Kuppler, R. J.; Zhou, H. C., Selective Gas Adsorption and Separation in Metal-Organic Frameworks. Chem. Soc. Rev. 2009, 38 (5), 1477-1504.

[53] Jung, Y., Multiple predicting K-fold Cross-Validation for Model Selection. J. Nonparametr. Stat. 2018, 30 (1), 197-215.
